# The Dose-Dependent Effect of Carbon Quantum Dots as a Photosynthesis Enhancer on Soybean Plant Growth

**DOI:** 10.3390/nano15201603

**Published:** 2025-10-21

**Authors:** Qianyuyue Wang, Kun Lv, Jian Song, Moyan Li, Xingnan Ouyang, Chengcheng Liu, Shuang Gong, Jinxing Wang, Jianming Li, Zhe Zhang

**Affiliations:** 1 College of Horticulture, North West Agriculture and Forestry University, Yangling, Xianyang 712100, China; wqyy@nwafu.edu.cn (Q.W.); ivkun@nwafu.edu.cn (K.L.); c19076898800@163.com (J.S.); limoyan@nwafu.edu.cn (M.L.); ouyangxn@nwafu.edu.cn (X.O.); liuchengcheng@nwafu.edu.cn (C.L.); gongshuang@nwafu.edu.cn (S.G.); lijianming66@nwsuaf.edu.cn (J.L.); 2 Technological Institute of Materials & Energy Science, Xijing University, Xi’an 710123, China

**Keywords:** nanomaterials, carbon quantum dots, dose-dependent, agriculture, soybean

## Abstract

When carbon quantum dots (CDs) are used to enhance photosynthesis, they inevitably enter the plant. However, the dose-dependent effects of CDs on plant growth are poorly understood. In this study, we investigated the dose-dependent effects of CDs on soybean growth. CDs were synthesized from citric acid and urea via a hydrothermal procedure. The analysis of the structure, chemical composition, and optical properties revealed that synthetic CDs have a sphere-like shape with rich hydrophilic groups on their surface. CDs exhibited superior upconverted photoluminescence properties and emitted strong fluorescence (exciting wavelength of 220 nm; emitting wavelength of 438 nm). Various concentrations of synthetic CDs (0–1000 mg L^−1^), as a photosynthesis enhancer, were applied to soybean plants under hydroculture for 1–10 days. CDs positively affected soybean growth at concentrations less than 200 mg L^−1^. However, at higher concentrations (500 or 1000 mg L^−1^), they exhibited significant toxicity to plant growth, which was evidenced by the mass accumulation of CDs in damaged leaves. Metabolomic and transcriptomic analyses indicated that CDs at a low concentration (100 mg L^−1^) could increase antioxidant and biomass accumulation in soybeans to promote plant growth. This study provided valuable insights into the impacts of CDs on plants in sustainable agricultural practices involving the use of nanomaterials.

## 1. Introduction

Carbon quantum dots (CDs) can promote light capture by photosystems in plants. Because of their ability to perform both down- and upconversions as an effective light promoter through tunable uptake of light, their application has shown promising results in enhancing photosynthesis [1]. Therefore, CDs have been used as a key nanomaterial to boost plant photosynthesis. CDs can be delivered through the roots or leaves via various methods, such as foliar spray [2], soil drenching, seed coating, and vacuum infiltration [3].

CDs have a series of qualities, such as small particle size, strong photoluminescence, strong photochemical stability, good water solubility, and high biocompatibility, which makes their application in the biological field very good, and they are mostly used as biosensors, photoelectricity catalysis, and so on. In recent years, due to the environmentally friendly nature of CDs, their application in the field of agriculture and development has also increased significantly. CDs can be synthesized by a variety of methods, which can be mainly categorized into two major groups: “top-down” and “bottom-up”. Top-down synthesis methods are generally used to prepare CDs by breaking down large carbon materials into nanoscale sizes, while top-down synthesis methods include chemical oxidation, electrochemical methods, and laser ablation. CDs prepared using citric acid and urea as precursors using hydrothermal synthesis have been extensively studied [4,5,6]. Previous studies have consistently reported that applying CDs in agriculture may be significantly beneficial for improving agricultural sustainability, as they can enhance crop growth and yield. However, there are concerns about their potential risks to human health and the environment. It is well known that nanomaterials can act as a double-edged sword; they may be toxic to environment and humans because of their small size and unique properties, facilitating their entry and accumulation in the environment and living organisms [7,8,9]. For example, the accumulation of nanomaterials in plants and their potential entry into the food chain raise concerns about their potential impact on human health [10]. Additionally, the interaction of nanomaterials with soil components can significantly negatively affect soil microbial communities, which could subsequently affect soil health and ecosystem functioning [11].

Although the appropriate concentration of CDs for agricultural use has not been assessed, it can be inferred from previous studies that the migration, distribution, and toxic effects of nanomaterials in living organisms are dose-dependent. For instance, previous studies have demonstrated significant differential effects of metal nanoparticles at 50 mg/L concentrations: Mn_3_O_4_ and Fe nanoparticles were shown to enhance the maximum quantum yield (by 23%) and ATP synthesis (by 43%) in spinach mesophyll protoplasts, while equivalent concentrations of Ag and MoS_2_ nanoparticles inhibited photosynthetic activity, with Ag NPs reducing whole-plant biomass by 24%, highlighting the critical importance of both the material composition and concentration [12]. Similarly, noble metal nanocrystals (e.g., Au/Ag) can enhance the photocurrent through plasmon resonance effects; however, higher concentrations may compromise system quantum yield due to energy transfer losses, suggesting potential risks associated with excessive application [13]. In contrast, far-red carbon dots (FR-CDs) at low concentrations demonstrated both efficacy and biosafety, converting UV light to photosynthetically active far-red radiation (625–800 nm), which increased the electron transport rate in lettuce by 28% while boosting fresh and dry weights by 51.14% and 24.60%, respectively, without observed toxicity, thereby providing a model for dose optimization of agricultural nanomaterials [14]. Therefore, it can be inferred that an appropriate concentration of CDs, under a certain threshold value, may be usable in agriculture to ensure a positive impact on plant growth [15]. Nevertheless, high concentrations of CDs may negatively impact plant growth. CDs can address the challenges of increasing overall agricultural production via sustainable pathways, providing high yields and promoting environmentally accepted agricultural practices (the efficient use of water, the maintenance of soil fertility, and minimal agrochemical pollution) [16]. However, the dose-dependent effects of CDs on agricultural systems should be fully studied to develop strategies to minimize their potential risks while maximizing their potential benefits. However, studies simultaneously assessing the effect of CD applications on plant growth and their toxicity are scarce.

In this study, CDs were successfully prepared via a hydrothermal procedure, and the microstructural morphology and physicochemical and optical properties of CDs were investigated. Further, the CDs were allowed to diffuse into soybean plants under hydroculture cultivation, and their subsequent dose-dependent (0–1000 mg L^−1^) effects on soybean growth were studied. The physiological response of soybean to various CD concentrations was assessed. The genetic regulation and molecular mechanisms underlying the effects of CDs on soybean were explored using metabolomic and transcriptomic analyses. To the best of our knowledge, this is the first study to investigate the accumulation and dose-dependent effect of CDs in plants. Our results provided a foundation for enhancing the safety and sustainability of CDs applications in agricultural practices and provided a reference for regulating and minimizing the potential risks of CDs in future agricultural applications.

## 2. Materials and Methods

### 2.1. Materials

Urea and citric acid were purchased from Aladdin (Shanghai, China). Other chemicals were purchased from Tianjin Fuchen Chemical Reagent Co., Ltd., Tianjin, China. All reagents were of analytical grade.

### 2.2. Synthesis of CDs

CDs were synthesized using a simple hydrothermal method. Briefly, a mixture of 10 g of urea, 30 g of citric acid, and 100 mL of distilled water was transferred into a Teflon-lined stainless steel high-pressure reaction autoclave. The autoclave was tightly sealed and heated in an oven at 180 °C for 5 h. Upon reaching room temperature, the mixture underwent centrifugation at 8000 rpm for a duration of 50 min to eliminate larger particulates. The supernatant was then filtered through a 0.22 μm membrane filter, after which the resulting filtrate was placed into a 1000 Da molecular weight cut-off dialysis bag and dialyzed for 12 h, with the deionized water being replaced at 6 h intervals. Subsequently, the dialyzed solution within the dialysis bag was retrieved. Through freeze-drying, aqueous components were removed from the CDs suspension. The synthesized CDs were ultimately preserved under dry conditions at temperatures not exceeding 4 °C, awaiting subsequent characterization and practical use.

### 2.3. Characterization Methosds of CDs

The morphology of CDs was observed using transmission electron microscopy (TEM, Talos F200S, USA). The samples were diluted using deionized water, and the diluted CD solution was applied dropwise onto a copper grid using a micropipette. The TEM grid on which the samples were dropwise applied was vacuum-dried to ensure that there was no residual solvent in the samples, and the dried samples were fixed in a sample holder and loaded into the TEM apparatus for testing.

The crystalline phases of CDs were examined using X-ray diffractometer (XRD, Bruker D8 Advance, Holland; X’pert diffractometer using Cu-Kα radiation) with a scanning speed of 0.03° (2θ) s^−1^. The CDs were uniformly coated and dried on glass or silicon wafers to ensure uniform distribution of the samples. The scanning angle range was adjusted (5–85°). An XRD scan was performed, and the diffraction peak positions were recorded. The crystalline phase structure of the CDs material was determined by comparing the diffraction angle and peak positions.

Additionally, the elemental state of CDs was examined using an X-ray photoelectron spectrometer (XPS, K-ALPHA, UK) at monochromatic Al Kα radiation (150 W, 15 kV, 1486.6 eV). The samples of CDs were uniformly deposited onto the conductive substrate to ensure uniform drying. Full and fractional spectral scans were made. Functional groups (e.g., C-N, C=O, etc.) were identified by observing the positions of binding energy peaks through the dedicated software Avantage, V6.7 and the relative contents of different functional groups were calculated.

The functional groups of CDs were identified using FTIR spectra (Fourier transform infrared spectroscopy, Nicolet NICOLET IS50, USA) in transmission mode using powder and KBr films (1%). All spectra were recorded within the range of 4000–500 cm^−1^ at q resolution of 4.0 cm^−1^. The CDs were ground into powder with KBr in the ratio of 1:100 before determination, and KBr-pressed tablets were made for FTIR spectroscopy. FTIR spectral analysis was performed covering the wavenumber range from 4000 to 500 cm^−1^ with a spectral resolution of 5.0 cm^−1^.

Raman spectral analysis of the as-prepared carbon dots was performed over the 400–4000 cm^−1^ range employing a Ram HR Evolution spectrometer (Andor SR-500i, UK), with excitation provided by a 532 nm. The samples of CDs were uniformly deposited on slides and dried, and the Raman spectra were scanned by selecting a laser wavelength of 325 nm to identify the structure of the materials by the D and G peaks.

The UV-Vis absorption spectra of CD solutions were determined in the 200–1000 nm range using a UV-Vis spectrophotometer (Shimadzu UV-2550 UV-Vis, Japan). Using a Shimadzu F-7000 fluorescence spectrophotometer (Japan), emission spectra were recorded between 310 and 460 nm using a standard long-pass filter to eliminate scattered excitation light, and the slit widths of both excitation and emission light were 1.5 nm for fluorescence measurements, with the fluorescence intensity being determined from the y-axis values of the PL spectra. The test was performed with a special cuvette after the sample was fully dissolved in deionized water (concentration of 1 mg/mL).

### 2.4. Cultivation Experiment

#### 2.4.1. Soybean Cultivation and Phytotoxicity Testing

To investigate the possible toxicity of CDs on soybean growth, soybean seedlings were cultivated under various concentrations of CDs (0, 50, 100, 200, 500, and 1000 mg L^−1^). The optimal concentration of CDs for boosting photosynthesis was identified by comprehensively evaluating photosynthetic and growth indexes.

In brief, the selected soybean seeds were first soaked in ultrapure water at 50 °C for 15 min. Further, the soybean seeds were sown in a hydroponic seedling tray (24.5 cm × 32.5 cm × 4.5 cm). The seed trays were covered with humidity domes and sprayed with ultrapure water daily to prevent desiccation during germination. The domes were subsequently removed 4 days after sowing. The seeds were germinated and produced aquatic root system after cultivation at 22 °C, 18 h/6 h light/dark photoperiod, and illuminance of approximately 1800 *lx*. The seeds with a growth length of 5–7 cm were selected and transferred into a hydroponic tank (305 cm × 185 cm × 140 cm) and fixed in a fixing basket using fixing cotton. The seedlings were watered with Hoagland nutrient media to supply the nutrients. After 10 days, the seedlings were hydroponically cultured in a vial filled with Hoagland nutrient media (80 mL) consisting of different concentrations of CDs (0–1000 mg L^−1^), with the entire vessel wrapped in aluminum foil during the cultivation. Each vial comprised five soybean seedlings, and the experiment included six replicates. Using a illumination incubator, soybean seedlings were maintained for a 10-day period with 12 h photoperiodic illumination (30,000 *lx*) at 25 ± 2 °C. The samples were collected at 5 and 10 days. The harvested plants were washed under ultrasonication in ultrapure water and divided into roots, stems, and leaves for further analysis. For metabolomic and transcriptomic studies, leaf samples were sequentially rinsed with ultrapure water and PBS solution, flash-frozen in liquid nitrogen, and preserved at −80 °C.

#### 2.4.2. Assessment of Photosynthetic and Growth Indexes

The net photosynthesis rate (P_n_), stomatal conductance (C_d_), intercellular CO_2_ concentration (C_i_), and transpiration rate (T_r_) of soybean leaves were measured using a LI-6800 portable photosynthetic analyzer (Lincoln, USA). A leaf with normal function was selected from each group. Measurements were taken once every 2 h and repeated three times each time. Statistical analysis was performed by calculating the mean values with standard deviations from triplicate measurements.

The leaves were kept in dark for 20 min, and further, the maximum photochemical rate (*F_v_*/*F_m_*) was measured using a high-resolution chlorophyll fluorescence imager (Hexagon-Imaging-PAM, Germany). Confocal images of chloroplasts were obtained with laser excitation wavelength of 405 nm and emission wavelength of 700–790 nm.

The contents of nutrient elements (N, P, and K) in the root, stem, and leaf were measured using a standard method [17].

The harvested leaves were imaged using a Leica stereo fluorescence microscope (M205FCA, Germany). For confocal imaging, CD-treated leaves were placed on a glass slide with the backside facing upward. The laser excitation wavelength used was 230 nm, and the emission fluorescence was monitored from 400 to 550 nm.

#### 2.4.3. Analysis of Metabolites and Transcriptome of Soybean Leaves

The metabolites and transcriptome of soybean leaves exposed to 0 (control) or 100 mg L^−1^ CDs for 10 days were analyzed. The specific methods can be found in the literature reports [18].

## 3. Results and Discussion

### 3.1. Characterization of CDs

The key properties of CDs, including their morphological and physicochemical properties, are highly correlated with their diffusivity, uptake, and translocation in plants.

**Figure 1:**
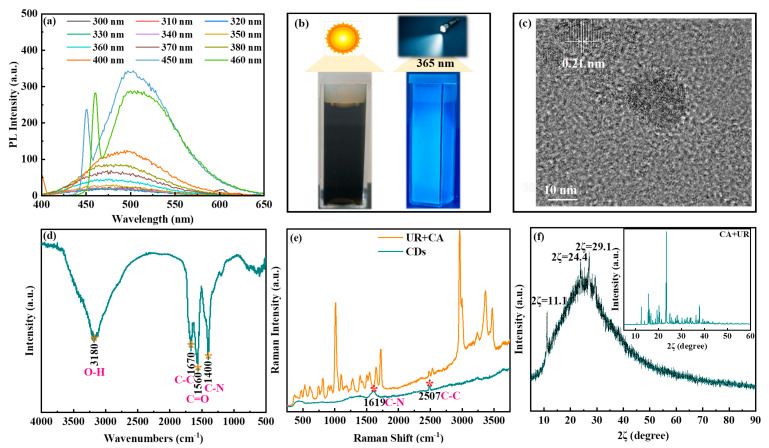
The PL spectra of CDs (**a**). The photographs of CDs under sunlight and 365 nm UV-light (**b**). The high-magnification TEM image of CDs (**c**). Infrared absorption spectrum with Fourier transform of CDs (FTIR) (**d**) and Raman spectra of the CDs (**e**). XRD test patterns of CDs and the mixing of urea and citric acid (**f**). The FTIR, Raman, and XRD spectra were analyzed to further confirm the structural information of CDs. The FTIR spectra of CDs are shown in (**d**). The peak around 3180 cm^−1^ (the stretching vibrations of the O-H group) and that at 1670 cm^−1^ (the C-C bending vibrations) from citric acid were observed in the CDs [21]. The peaks at 1560 and 1400 cm^−1^ were assigned to the C=O and C-N stretching vibrations [22], respectively. The Raman spectrum of CDs was completely different from that of the mixture of urea and citric acid (**e**). Compared with the mixture of urea and citric acid, new peaks appeared at 1619 (C-N) and 2507 (C-C) cm^−1^ in the Raman spectrum of CDs. In the XRD pattern of CDs (**f**), the initial characteristic peaks of the mixture of urea and citric acid disappeared (inset in (**f**)), and new diffraction peaks were observed around 2θ of 11°, 24°, and 29° in the XRD pattern of CDs. This variation could potentially originate from distinct surface functional group compositions, where the C-N bonding might have contributed to the observed minor crystalline structural modifications.

Figure 1a shows the fluorescence spectra of 1 mg mL^−1^ CDs. The top three excitation wavelengths of CDs were 320, 400, and 460 nm according to the fluorescence intensity. This was consistent with the results observed under UV irradiation; 1 mg mL^−1^ CDs dissolved in water emitted a visually detectable blue fluorescence when exposed to a 365 nm UV lamp. At the same time, the UV-Vis spectra (200–1000 nm) of the same concentration of CDs solution revealed strong absorption at the excitation wavelength (365 nm), consistent with the fluorescence behavior. This correlation confirms the optical activity of the CDs at this concentration (Figure 1b and Figures S2 and S3). These results revealed the superior photoluminescence property of CDs [19]. The maximum emission peak of CDs was observed at 438 nm under an excitation peak at 220 nm, originating from the π-π* transition of aromatic sp^2^ conjugate domains (C=C, C-C) and n-π* transitions of the -CONH- bond in aromatic structures [20]. The CDs were present as dispersions, and the TEM image displayed distinct lattice fringes with a distance of 0.21 nm and their size of approximately 10–20 nm (Figure 1c and Figure S1).

**Figure 2:**
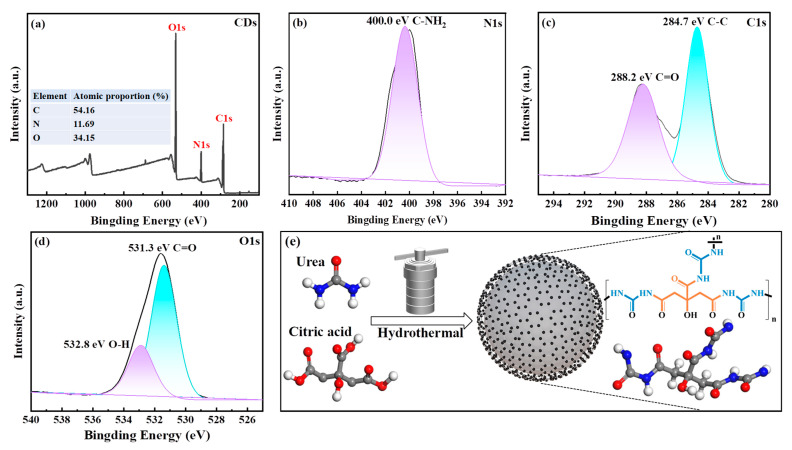
XPS spectra of CDs. The full scan survey spectrum (**a**). High-resolution XPS spectrum of N 1s (**b**), C 1s (**c**), and O 1s (**d**). The schematic synthesis of CDs (**e**). The possible mechanism of CD formation could be inferred as the bonding between hydroxyl groups of citric acid and amino groups of urea, which can be supported by the FTIR, Raman, and XPS spectra analyses (Figure 1d–f). The peaks at 1400 cm^−1^ were associated with C-N stretching vibration that occurred in FTIR, which was consistent with the results of Raman, XPS, and XRD spectra analyses. This indicated the formation of a link between nitrogen from urea and carbon from citric acid in the crystalline phases of CDs. As reported in a previous study, citric acid initiates a dehydration process in the presence of NH_3_ at high temperatures, generating pyridine-ring-based fluorophore species [25]. The predominance of -CO and -OH groups on the surface of CD samples endows excellent water solubility and photoluminescence properties.

XPS spectra (Figure 2) indicated that CDs contained C (54.16%), N (11.69%), and O (34.15%) elements. The prominent peaks in CDs were recognized as follows: C1s peaks at 285 eV, N 1s peaks at 400 eV, and O 1s peaks at 531 eV (Figure 2a). The high-resolution N 1s XPS spectrum of CDs contained one Gaussian peak at 400.4 eV that aligned with C-N (Figure 2b) [23]. Peak fitting of the C1s XPS spectrum (Figure 2c) revealed two Gaussian peaks at 284.7 and 288.2 eV. The sp^2^ carbon (C-C/C=C) in citric acid, sp^3^ carbon (C-O), and C=O in the carboxyl group were represented by these peaks [2]. Moreover, the binding energies of 531.3 and 532.8 eV were the two main signals for O 1s, corresponding to C=O and O-H [24].

### 3.2. Effect of CDs on Soybean Growth

**Figure 3:**
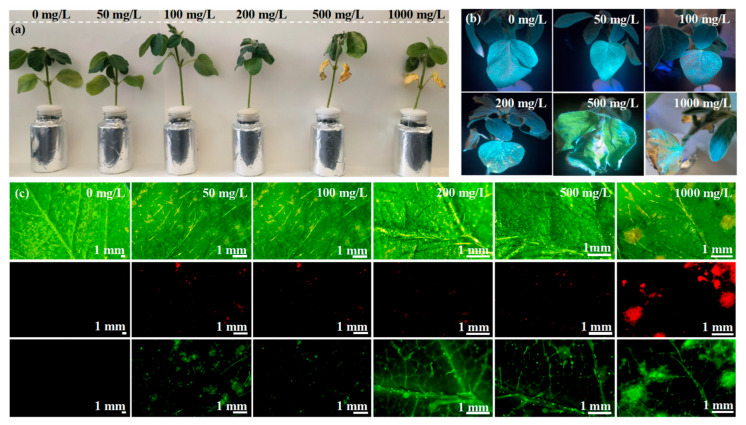
Effects on the growth of CDs in soybeans. Growth states at different CD exposure concentrations (**a**). The distribution of CDs in soybean leaves (**b**,**c**). The effect of various concentrations of CDs on plant photosynthesis was investigated by assessing the values of P_n_, C_d_, C_i_, and T_r_. Various concentrations of CDs did not remarkably influence photosynthesis on the 1st day of cultivation, likely due to the less absorption of CDs in soybean on the 1st day (Figure S4). After 5 days of cultivation, the P_n_ was low (9.43–9.48 μmol CO_2_ m^−2^ s^−1^) at 0 and 50 mg L^−1^ of CDs (Figure 4a). However, the photosynthetic activity significantly increased after exposure to 100 mg L^−1^ of CDs; compared with the control, P_n_, C_d_, C_i_, and T_r_ increased by 30.6%, 18.8%, 45.0%, and 76.8%, respectively (Figure 4a,b,d). However, higher CD concentrations (500 or 1000 mg L^−1^) exhibited opposite results, with significantly lower P_n_ (6.76 ± 0.57 and 2.93 ± 0.33 μmol CO_2_ m^−2^ s^−1^, respectively) and C_d_ (0.12 ± 0.002 and 0.06 ± 0.001 mol H_2_O m^−2^ s^−1^, respectively) than the control (9.43 ± 0.59 μmol CO_2_ m^−2^ s^−1^ and 0.16 ± 0.003 mol H_2_O m^−2^ s^−1^, respectively). These results implied that higher CD concentrations (500 or 1000 mg L^−1^) significantly inhibited photosynthesis in soybean. This could be attributed to two factors. First, the local accumulation of CDs in soybean leaves blocked the vascular structure and thus inhibited photosynthesis through shading, and second, CD accumulation disturbed the electron and energy transfer in the photosystem. At 100 mg L^−1^ of CDs, C_i_ increased compared with that under 0 or 50 mg L^−1^ of CDs (Figure 4c). C_i_ was 351.38 ± 1.69, 327.23 ± 2.54, 330.42 ± 2.30, and 341.51 ± 2.68 μmol CO_2_ mol^−1^ under 100, 200, 500, and 1000 mg L^−1^ of CDs, respectively, slightly higher than that observed at 0 and 50 mg L^−1^ of CDs (242.42 ± 1.18 and 256.28 ± 2.77 μmol CO_2_ mol^−1^, respectively). Consistent with other photosynthetic indexes, C_i_ was the highest at 100 mg L^−1^ of CDs, which may be the result of enhanced maximum solar energy conversion efficiency of the photosystem II complex in the chloroplasts at 100 mg L^−1^ CDs [27]. This was further supported by the results of *F_v_*/*F_m_* ratio (Table 1). The estimated *F_v_*/*F_m_* ratio was 0.725 ± 0.008 in the control. The *F_v_*/F*_m_* ratio slightly increased to 0.728 ± 0.004, 0.808 ± 0.011, and 0.748 ± 0.009 with 50, 100, and 200 mg L^−1^ of CDs, respectively, and reduced to 0.693 ± 0.006 and 0.697 ± 0.004 with 500 and 1000 mg L^−1^ of CDs. The enhancement in *F_v_*/*F_m_* ratio in soybean leaves after exposure to CD concentration ≤ 200 mg L^−1^ was likely due to the CD-mediated conversion of UV light (unavailable light for the chloroplasts) irradiated on the chloroplast surface to the light in the range of 40–700 nm (available light for the chloroplast). The most effective UV light conversion efficiency was observed at the CD concentration of 100 mg L^−1^.

Figure 3a displays the phenotypic changes in soybean after exposure to various CD concentrations for 10 days. The color of soybean leaves remained green when the CD concentration was ≤200 mg L^−1^, whereas the soybean leaves turned yellow when the CD concentration reached 500 mg L^−1^ (Figure 3a). Therefore, CDs exhibited dose-dependent effect on soybean plants. Soybean growth exhibited no sign of suppression at CD concentrations ≤ 200 mg L^−1^; however, CDs exhibited considerable phytotoxicity to soybean at 500 and 1000 mg L^−1^ when the leaves were significantly damaged (Figure 3b). A stereofluorescence microscope was used to investigate the distribution of CDs in soybean leaves (Figure 3c). Fluorescence was not detected in the leaves in the control (0 mg L^−1^ CDs) and was rarely detected in the leaves in healthy soybean leaves when the CD concentration was ≤200 mg L^−1^. However, intense fluorescent spots in the images of CD-treated leaves at 500 and 1000 mg L^−1^ indicated that CDs accumulated in soybean leaves. As per a previous study, the nanoparticles can pass through cracks in the root system, subsequently internalizing into the cytoplasm through endocytosis by the cell membrane [26]; a large percentage of nanoparticles accumulate in the roots and migrate to the stems, further migrating to the leaves.

**Figure 4:**
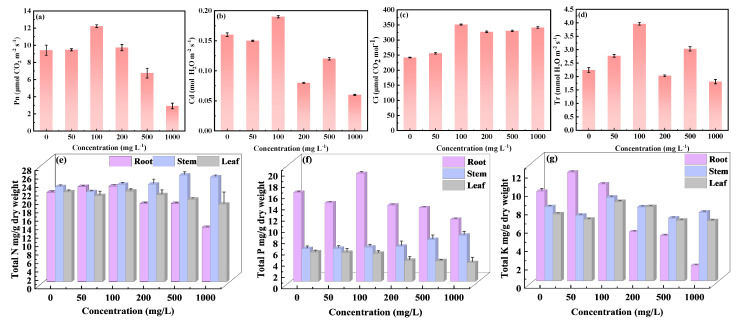
Photosynthetic parameters: the net photosynthesis rate (P_n_) (**a**), stomatal conductance (C_d_) (**b**), intercellular CO_2_ concentration (C_i_) (**c**), and transpiration rate (T_r_) (**d**) of soybeans cultured for 5 days under different CDs concentrations; N (**e**), P (**f**), and K (**g**) contents in soybean roots, stems, and leaves after exposure of 10 days and different exposure concentrations.

The contents of macroelements (N, P, and K) were measured in soybean roots, stems, and leaves after exposure to CDs for 10 days (Figure 4e–g). The N, P, and K contents in soybean roots and leaves exhibited the same trend. They were higher when the CD concentration was ≤200 mg L^−1^. This indicated that direct contact between the roots and high concentrations of CDs (>200 mg L^−1^) might damage the roots, further causing toxicity to soybean growth by limiting the ability to absorb nutrient elements. The local accumulation of CDs in soybean leaves blocked the vascular structure, thus probably limiting the absorption of nutrients. The stem was the direct transmission channel of CDs between soybean roots and leaves. The distribution of N, P, and K contents in the stem exhibited an inconsistent trend. The N and P contents of soybean stem gradually increased with CD concentrations, whereas the K content of soybean stem decreased at high CD concentrations (>200 mg L^−1^).

### 3.3. Metabolomic and Transcriptomic Profiles of Soybean Leaves After Exposure to CDs

**Figure 5:**
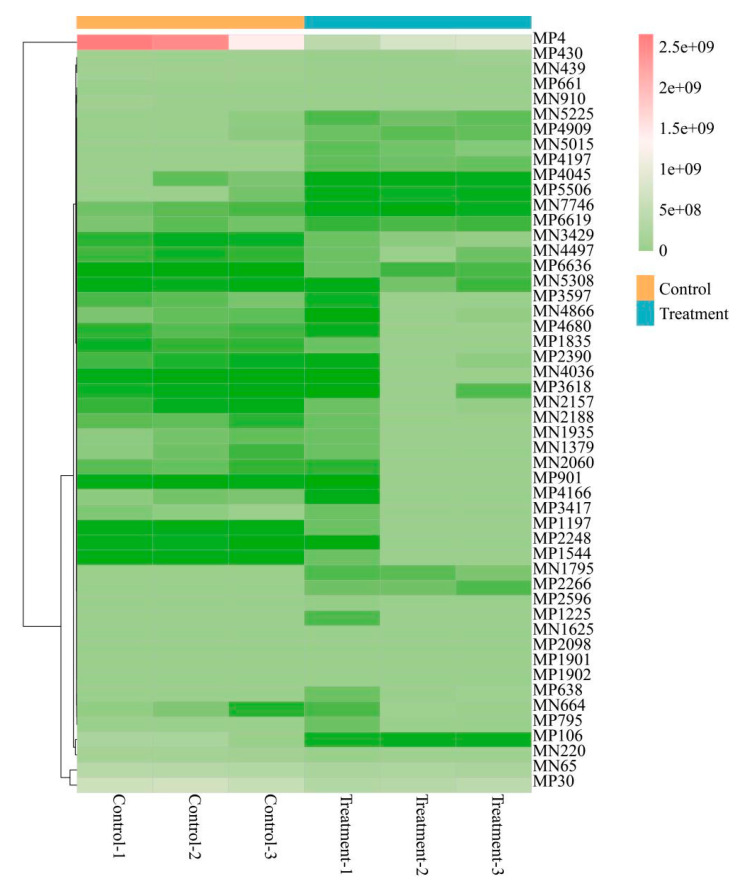
Hierarchical clustering analysis performed on the top 50 metabolites under the criteria of *p* value < 0.05. The upregulated metabolites were subjected to KEGG pathway enrichment analysis (Table S3). The significantly different pathways (*p* value < 0.05) between both groups were flavone and flavonol biosynthesis, isoflavonoid biosynthesis, flavonoid biosynthesis, terpenoid backbone biosynthesis, phenylpropanoid biosynthesis, biosynthesis of secondary metabolites, and porphyrin metabolism. In the 50 metabolites analyzed, the number of upregulated metabolites was higher than that of downregulated metabolites, and five metabolites (luteolin, formononetin-7-O-glucoside, formononetin-7-O-glucoside-6″-O-malonate, coniferol, and benzoic acid (Table S2)) were highly upregulated. Furthermore, the related genes (e.g., GLYMA_09G019900, GLYMA_04G220600, GLYMA_12G059100, GLYMA_05G124900, GLYMA_01G137700, GLYMA_14G156400, GLYMA_01G169200, GLYMA_11G243000, and GLYMA_14G195200) involved in the above metabolic pathways were characterized according to KEGG database (Table S4).

To elucidate the molecular mechanisms underlying the CD-enhanced photosynthetic performance, we conducted metabolomic profiling of soybean leaves. Orthogonal partial least squares-discriminant analysis (OPLS-DA) was employed to discriminate metabolic alterations of soybean leaves following treatment with 100 mg L^−1^ of CDs. The metabolites differed in two groups (exposed to 0 or 100 mg L^−1^ CDs) with R^2^ < 1 and *p* < 0.05 (Figure S5a). As evidenced by volcano plot analysis, distinct metabolic profiles were observed between CD-exposed and untreated samples, revealing CD-mediated metabolic alterations in foliar tissues. (Figure S5b). A total of 153 metabolites (76 significantly upregulated and 77 significantly downregulated) were quantified (*p* value < 0.05). The top-50 metabolites were selected as per the *p*-value ranking and were further analyzed using hierarchical clustering (Figure 5; the metabolites are listed in Supplementary Table S1). The observed metabolic changes induced by CDs in soybean leaves may be explained through transcriptional regulation analysis. Researchers identified significant alterations in the expression levels of growth-related metabolic genes, with comparative data showing distinct transcriptional profiles between CD-treated and control groups. (Figure S6), further confirming CDs’ metabolic influence through the identification of 4079 differentially expressed genes (2203 upregulated, 1876 downregulated, *p* < 0.05). Notably, the top-50 most significant top-50 up- and downregulated genes in each direction were systematically cataloged (Table S2).

**Figure 6:**
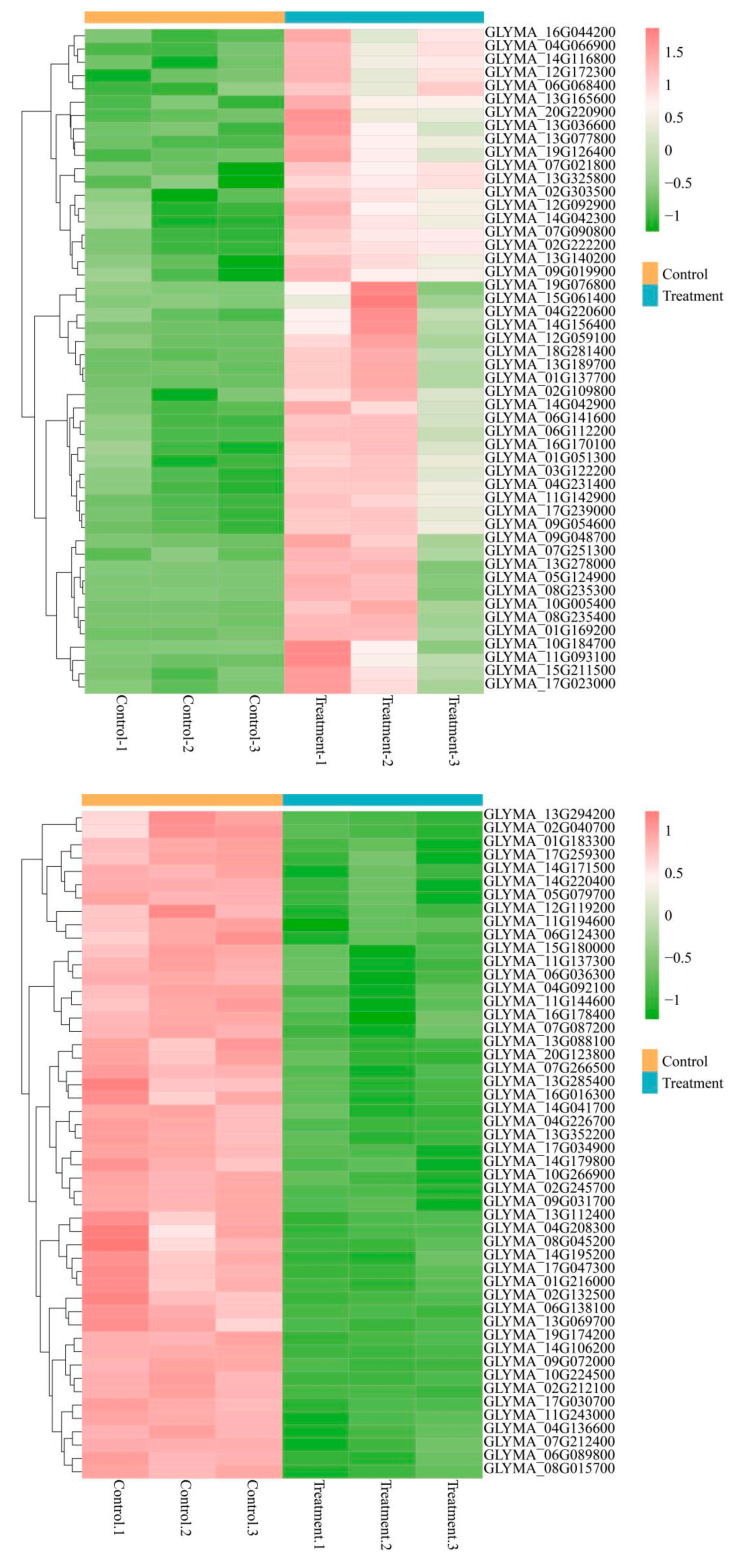
Hierarchical clustering analysis performed on the top 50 metabolites under the criteria of *p* value < 0.05.

The results of KEGG pathway enrichment analysis demonstrated that the CD-treated plants exhibited higher antioxidant and biomass accumulation (Figure 6). For example, flavone and flavonol biosynthesis were enhanced after exposure to 100 mg L^−1^ of CDs compared with the control. Flavones such as luteolin, phenylpropanoids, and terpenoids are the most widely distributed plant metabolites [28]. They can scavenge oxidative-damage-causing free radicals through inhibiting the activity of pro-oxidant enzymes that produce free radicals [29]. They act as signal regulators of various biochemical pathways in plant cells, playing critical roles in plant growth and adaptation to stress [30]. Isoflavonoids were significantly upregulated in the CD-treated group. Isoflavonoids are secondary metabolites of the phenylalanine pathway and are mainly found in the Leguminosae family [20]. They play an essential role in plant growth, development, and response to biotic and abiotic stresses [31]. Isoflavonoid synthase (EC 1.1414.87) is a membrane-bound cytochrome P450 monooxygenase, which is the key enzyme responsible for isoflavonoid biosynthesis (Figure S7). Simultaneously, the enhancement in flavonoid levels can enhance the growth of plants under stress conditions because of their capability of neutralizing reactive oxygen species [29,32]. Flavonoids, as polyphenolic secondary metabolites biosynthesized by plants, are critically involved in developmental regulation and plant defense systems against both oxidative stress and pathogenic microorganisms. Porphyrins participate in multiple fundamental biological processes, notably photosynthetic light harvesting, cellular signaling pathways, reactive oxygen species detoxification, and energy conversion mechanisms [33]. A slight increase in porphyrin levels in the CD-treated group was favorable for plant growth.

Phenylpropanoid biosynthesis, flavonoid biosynthesis, and isoflavonoid biosynthesis were significantly promoted after exposure to 100 mg L^−1^ CDs (Figure S7). The signaling pathways in soybean leaves were altered after exposure to CDs. This suggested that CDs at 100 mg L^−1^ can trigger signaling molecules, consequently changing the metabolism in plant leaves. After treatment with CDs, decreased oxidative stress and increased levels of secondary metabolites helped the plants to develop faster in the early growth stage and increase biomass. The changes in these biological pathways may account for the positive effect of CDs on soybean growth. (See Figure 6).

## 4. Conclusions

CDs are novel nanomaterials with scalable applications in agriculture. In this study, the dose-dependent effects of CDs on soybean plant growth were investigated. At a concentration of 100 mg L^−1^, the CDs treatment group increased the photosynthesis of soybeans by more than 30%. However, CDs positively affected plant growth at a low concentration (100 mg L^−1^), whereas high concentrations of CDs (500 or 1000 mg L^−1^) caused significant toxicity to the plants. Metabolomic and transcriptomic analyses indicated that 100 mg L^−1^ of CDs enhanced antioxidant activity and biomass accumulation in soybeans, promoting plant growth. Our results have implications for both agricultural applications and the environmental risk assessment of CDs. CDs can act as a double-edged sword; more efforts should be made in the future to gain insights into the dose-dependent effects of different types of CDs in different plants to fully understand the toxicity of CDs. Future efforts should focus on synthesizing lower-toxicity CDs using rational structural design and developing safer application methods to reduce the toxicity of CDs, such as introducing biodegradable functional groups into the structures of CDs or utilizing them in a controlled-release manner. Furthermore, the cause of the higher toxicity of CDs in higher concentrations has not been fully explored. CDs’ phagocytosis, migration, and transformation by plant cells, as well as their specific binding with cells, are topics for future research.

## Figures and Tables

**Table 1:** Statistical analysis of the *F_v_*/*F_m_* measured with dark adaptation for different samples (the results are presented as mean ± SD).

CD Concentration (mg L^−1^)	*F_v_*/*F_m_*
0	0.725 ± 0.008 c
50	0.728 ± 0.004 c
100	0.808 ± 0.011 a
200	0.748 ± 0.009 b
500	0.693 ± 0.006 d
1000	0.697 ± 0.004 d

## Data Availability

The original contributions presented in this study are included in the article/Supplementary Materials. Further inquiries can be directed to the corresponding author.

## Supplementary Materials

The supporting information can be downloaded at: https://www.mdpi.com/article/10.3390/nano15201603/s1.
