# Supplementary material for: The Dose-Dependent Effect of Carbon Quantum Dots as a Photosynthesis Enhancer on Soybean Plant Growth
Source: Nanomaterials (Basel). 2025 Oct 21;15(20):1603. doi: 10.3390/nano15201603 (PMC12566706; doi:10.3390/nano15201603)
Supplement: Supplementary file 1 [file nanomaterials-15-01603-s001.zip › nanomaterials-3589214-supplementary.pdf]

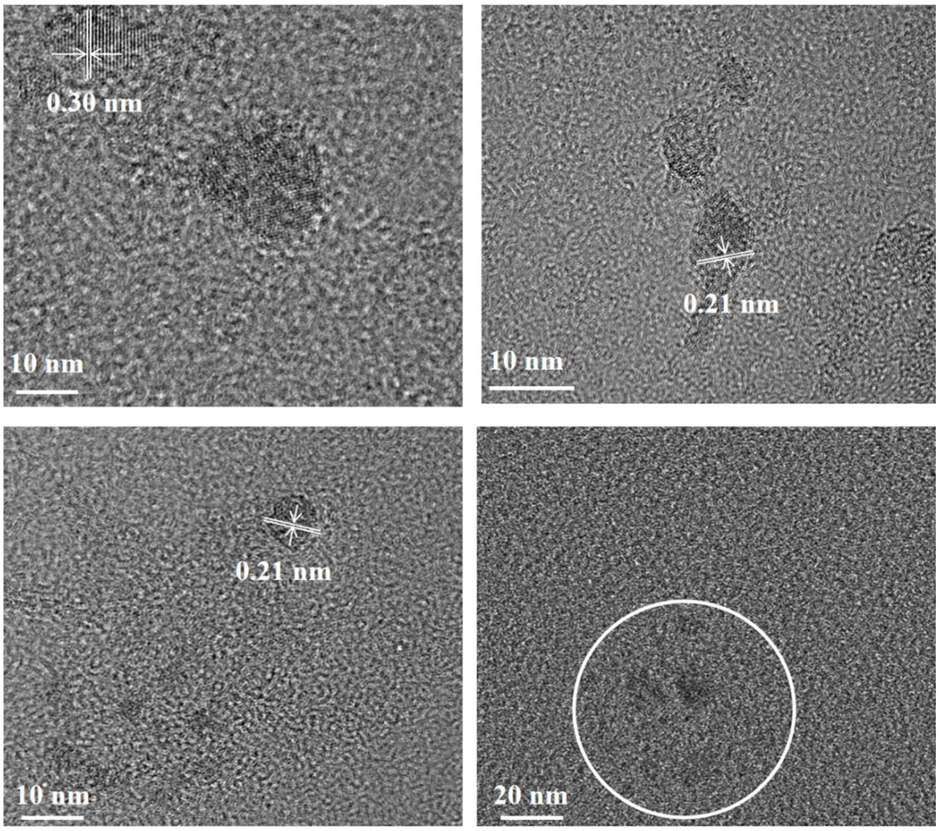

Figure S1. The high-magnification TEM image of CDs.

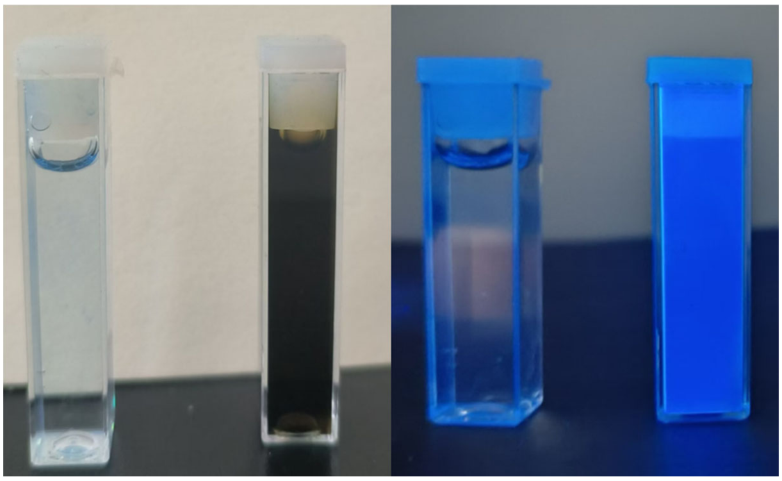

Figure S2. The photograph of the CDs under ambient lighting and under a UV lamp.

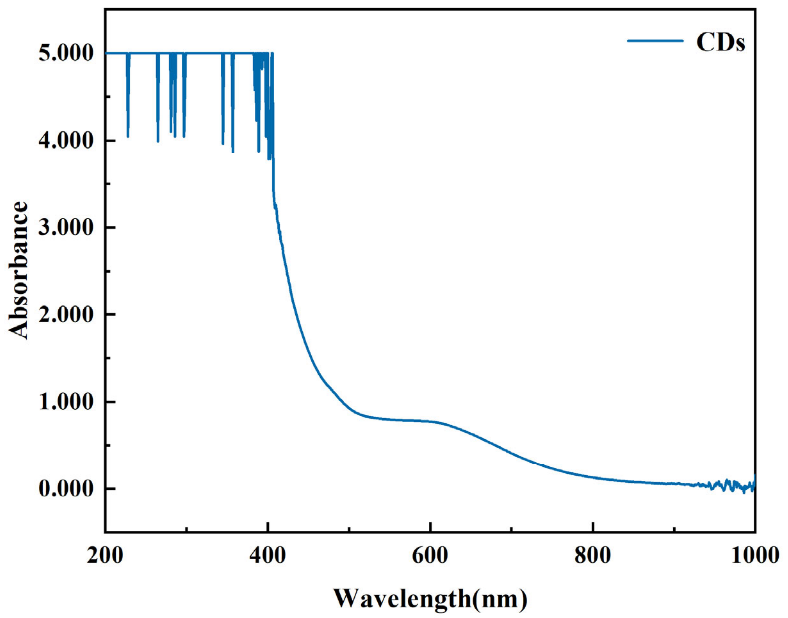

Figure S3. Absorbance values of CDs at wavelengths of 200-1000 nm.

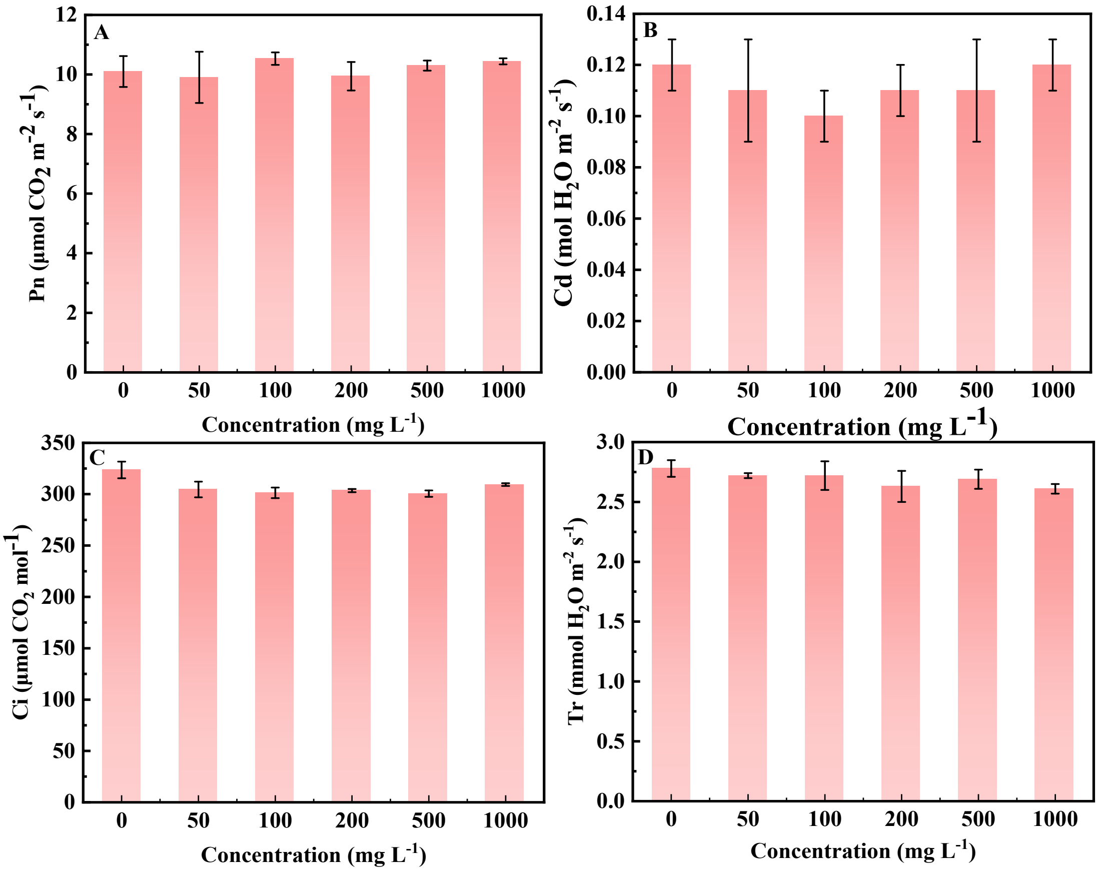

Figure S4. Photosynthetic parameters: the net photosynthesis rate (P_n), stomatal conductance (C_d), intercellular CO_2 concentration (C_i) and transpiration rate (T_r) of soybean were cultured for 1 days under different CD concentrations.

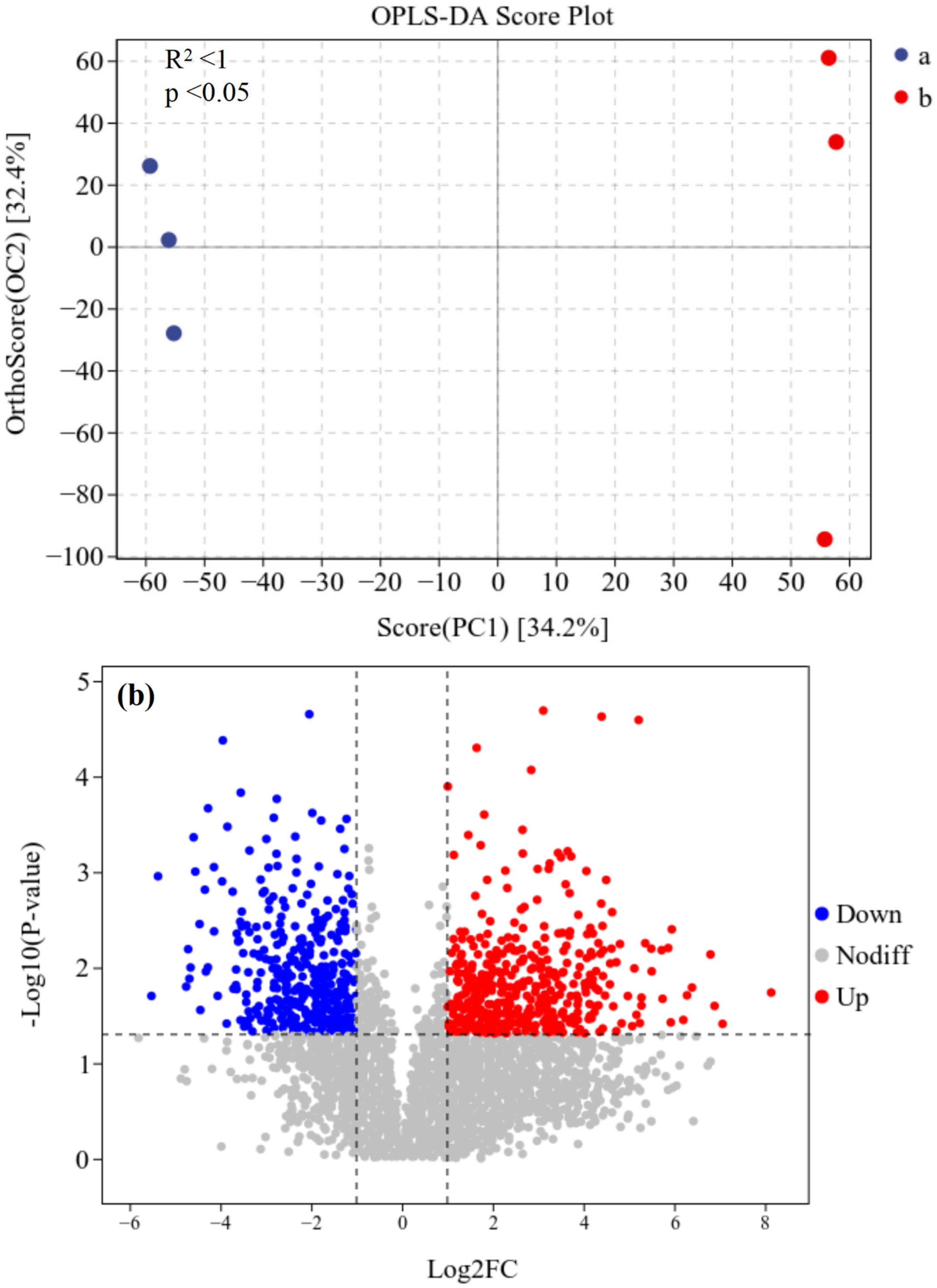

Figure S5. Orthogonal partial least-squares discriminant analysis (OPLS-DA) model (a) and volcano plot analysis (b) of differential metabolite analysis: a is control group (0 mg/L CDs), b is 100 mg/L CDs treatment group.

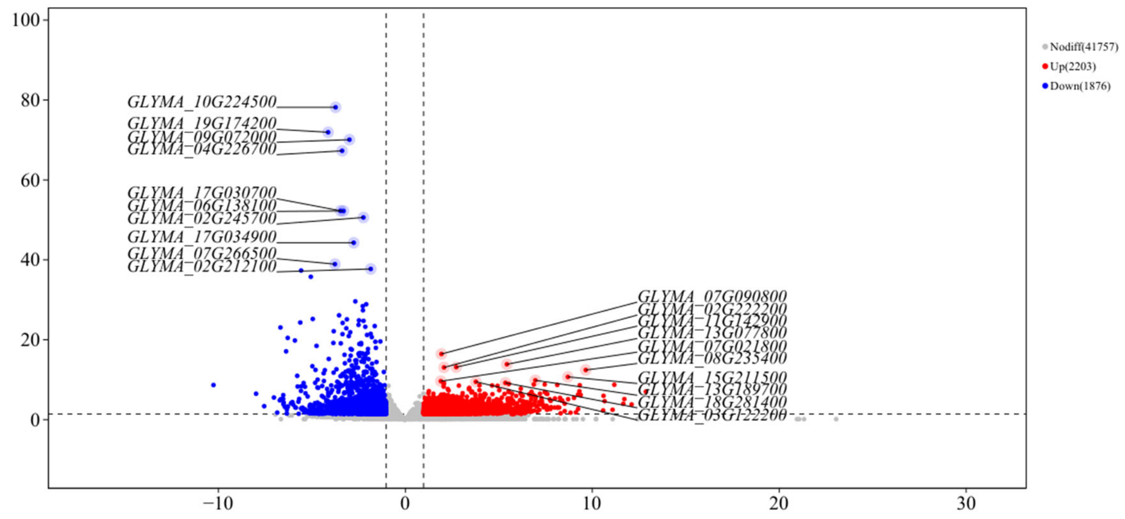

Figure S6. The volcano plot analysis of differential genes analysis.

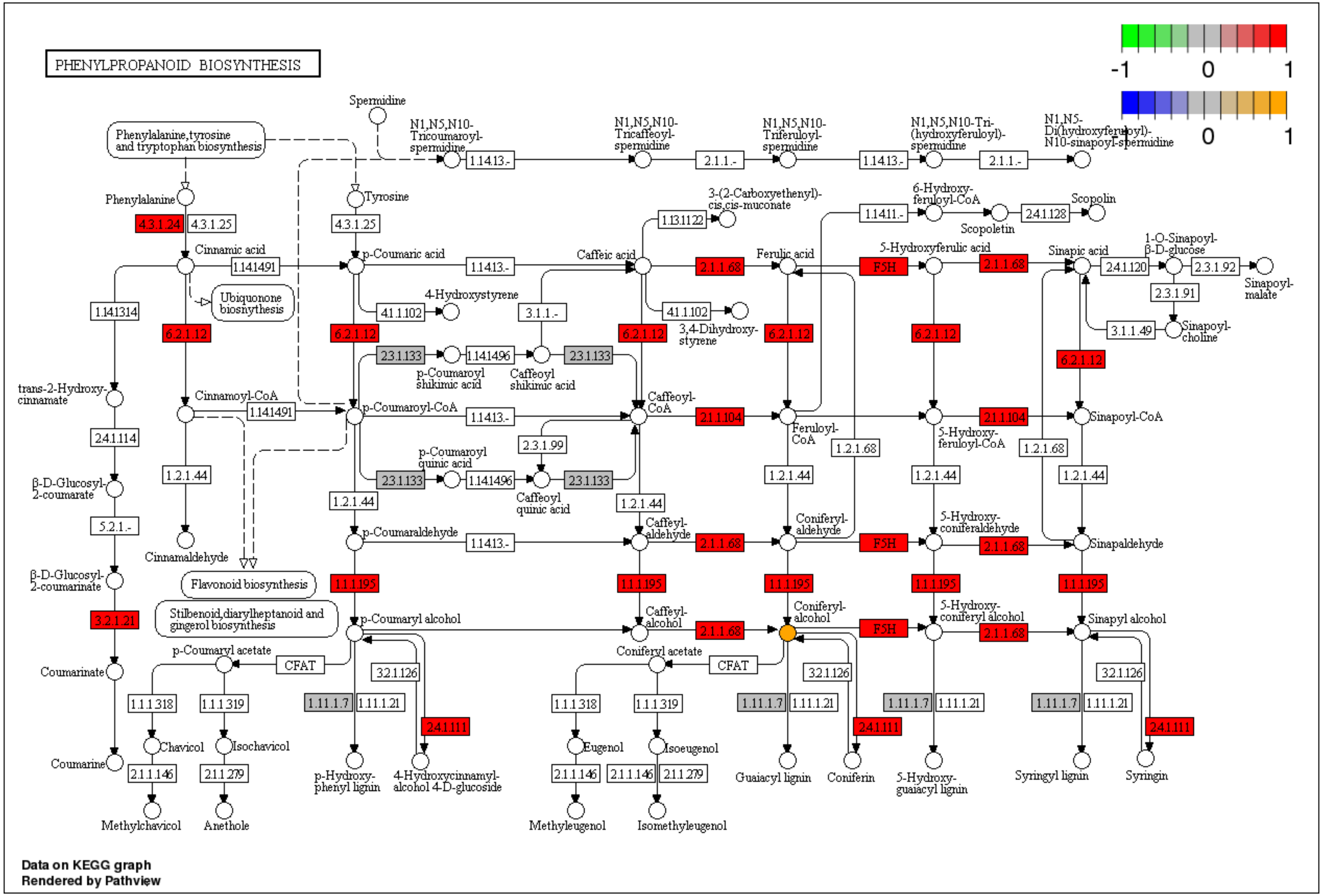
PHENYLPROPANOID BIOSYNTHESIS
-1
0
1
0
1
Spermidine
Phenylalanine,tyrosine and tryptophan biosynthesis
N1,N5,N10-Tricoumaroyl-spermidine
N1,N5,N10-Tricaffeoyl-spermidine
N1,N5,N10-Triferuloyl-spermidine
N1,N5,N10-Tri-(hydroxyferuloyl)-spermidine
N1,N5-Di(hydroxyferuloyl)-N10-sinapoyl-spermidine
1.14.13.-
2.1.1.-
1.14.13.-
2.1.1.-
Phenylalanine
Tyrosine
4.3.1.24
4.3.1.25
4.3.1.25
3-(2-Carboxyethenyl)-cis,cis-muconate
1.13.11.22
6-Hydroxy-feruloyl-CoA
1.14.11.-
Scopoletin
2.4.1.128
Scopolin
Cinnamic acid
1.14.14.91
p-Coumaric acid
1.14.13.-
Caffeic acid
2.1.1.68
Ferulic acid
F5H
5-Hydroxyferulic acid
2.1.1.68
Sinapic acid
2.4.1.120
1-O-Sinapoyl-β-D-glucose
2.3.1.92
Sinapoyl-malate
2.3.1.91
3.1.1.49
Sinapoyl-choline
1.14.13.14
Ubiquonone biosynthesis
4-Hydroxystyrene
4.1.1.102
3.1.1.-
4.1.1.102
3,4-Dihydroxy-styrene
6.2.1.12
6.2.1.12
6.2.1.12
6.2.1.12
6.2.1.12
6.2.1.12
2.3.1.133
1.14.14.96
2.3.1.133
p-Coumaroyl shikimic acid
Caffeoyl shikimic acid
trans-2-Hydroxy-cinnamate
Cinnamoyl-CoA
1.14.14.91
p-Coumaroyl-CoA
1.14.13.-
Caffeoyl-CoA
2.1.1.104
Feruloyl-CoA
2.1.1.104
Sinapoyl-CoA
5-Hydroxy-feruloyl-CoA
1.2.1.68
1.2.1.68
2.4.1.114
2.3.1.99
p-Coumaroyl quinic acid
2.3.1.133
1.14.14.96
2.3.1.133
Caffeoyl quinic acid
1.2.1.44
1.2.1.44
1.2.1.44
1.2.1.44
1.2.1.44
1.2.1.44
β-D-Glucosyl-2-coumarate
5.2.1.-
Cinnamaldehyde
p-Coumaraldehyde
1.14.13.-
Caffeyl-aldehyde
2.1.1.68
Coniferyl-aldehyde
F5H
5-Hydroxy-coniferaldehyde
2.1.1.68
Sinapaldehyde
β-D-Glucosyl-2-coumarinate
Flavonoid biosynthesis
1.1.1.195
1.1.1.195
1.1.1.195
1.1.1.195
1.1.1.195
3.2.1.21
Stilbenoid,diarylheptanoid and gingerol biosynthesis
p-Coumaryl alcohol
Caffeyl-alcohol
2.1.1.68
Coniferyl-alcohol
F5H
5-Hydroxy-coniferyl alcohol
2.1.1.68
Sinapyl alcohol
p-Coumaryl acetate
CFAT
Coniferyl acetate
CFAT
Coumarinate
3.2.1.126
3.2.1.126
3.2.1.126
1.1.1.318
1.1.1.319
1.11.1.7
1.11.1.21
1.1.1.318
1.1.1.319
1.11.1.7
1.11.1.21
1.11.1.7
1.11.1.21
1.11.1.7
1.11.1.21
2.4.1.111
2.4.1.111
2.4.1.111
Chavicol
Isochavicol
Eugenol
Isoeugenol
Coumarine
2.1.1.146
2.1.1.279
2.1.1.146
2.1.1.146
2.1.1.279
p-Hydroxy-phenyl lignin
4-Hydroxycinnamyl-alcohol 4-D-glucoside
Guaiacyl lignin
Coniferin
5-Hydroxy-guaiacyl lignin
Syringyl lignin
Syringin
Methylchavicol
Anethole
Methyleugenol
Isomethyleugenol
Data on KEGG graph
Rendered by Pathview

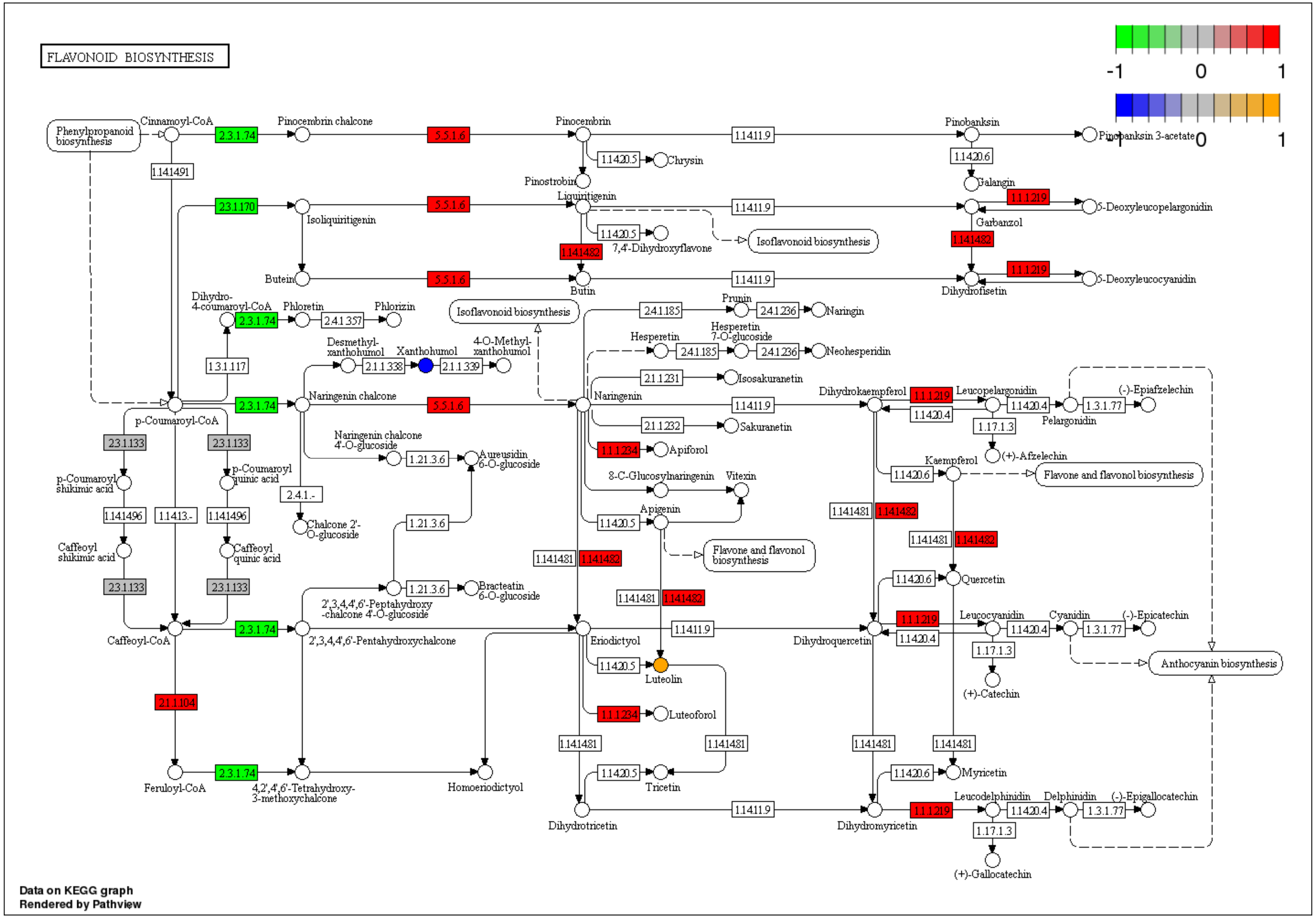

FLAVONOID BIOSYNTHESIS
-1 0 1
-1 0 1
Phenylpropanoid biosynthesis
Cinnamoyl-CoA
2.3.1.74
Pinocembrin chalcone
5.5.1.6
Pinocembrin
1.14.11.9
Pinobanksin
Pinobanksin 3-acetate
1.14.14.91
1.14.20.5
Chrysin
1.14.20.6
Pinostrobin
Galangin
2.3.1.170
Isoliquiritigenin
Liquiritigenin
1.1.1.219
5-Deoxyleucopelargonidin
Garbanzol
Isoflavonoid biosynthesis
1.14.14.82
7,4'-Dihydroxyflavone
Butein
Butin
Dihydrofisetin
5-Deoxyleucocyanidin
Dihydro-4-coumaroyl-CoA
Phloretin
2.4.1.357
Phlorizin
Prunin
2.4.1.185
2.4.1.236
Naringin
Hesperetin
Hesperetin 7-O-glucoside
Neohesperidin
1.3.1.117
Desmethyl-xanthohumol
Xanthohumol
4-O-Methyl-xanthohumol
2.1.1.338
2.1.1.339
2.1.1.231
Isosakuranetin
Dihydrokaempferol
Leucopelargonidin
(-)-Epiafzelechin
Naringenin chalcone
Naringenin
1.14.20.4
1.3.1.77
Pelargonidin
p-Coumaroyl-CoA
2.1.1.232
Sakuranetin
1.17.1.3
2.3.1.133
Naringenin chalcone 4'-O-glucoside
Aureusidin 6-O-glucoside
1.21.3.6
1.1.1.234
Apiforol
(+)-Afzelechin
Kaempferol
p-Coumaroyl shikimic acid
p-Coumaroyl quinic acid
8-C-Glucosylnaringenin
Vitexin
Flavone and flavonol biosynthesis
2.4.1.-
1.14.14.96
1.14.13.-
Apigenin
1.14.14.81
Chalcone 2'-O-glucoside
Caffeoyl shikimic acid
Caffeoyl quinic acid
Quercetin
2',3,4,4',6'-Peptahydroxy-chalcone 4'-O-glucoside
Bracteatin 6-O-glucoside
Caffeoyl-CoA
2',3,4,4',6'-Pentahydroxychalcone
Eriodictyol
Dihydroquercetin
Leucocyanidin
Cyanidin
(-)-Epicatechin
Luteolin
Anthocyanin biosynthesis
(+)-Catechin
2.1.1.104
Luteoforol
Myricetin
Feruloyl-CoA
4,2',4',6'-Tetrahydroxy-3-methoxychalcone
Homoeriodictyol
Tricetin
Leucodelphinidin
Delphinidin
(-)-Epigallocatechin
Dihydrotricetin
Dihydromyricetin
(+)-Gallocatechin
Data on KEGG graph
Rendered by Pathview

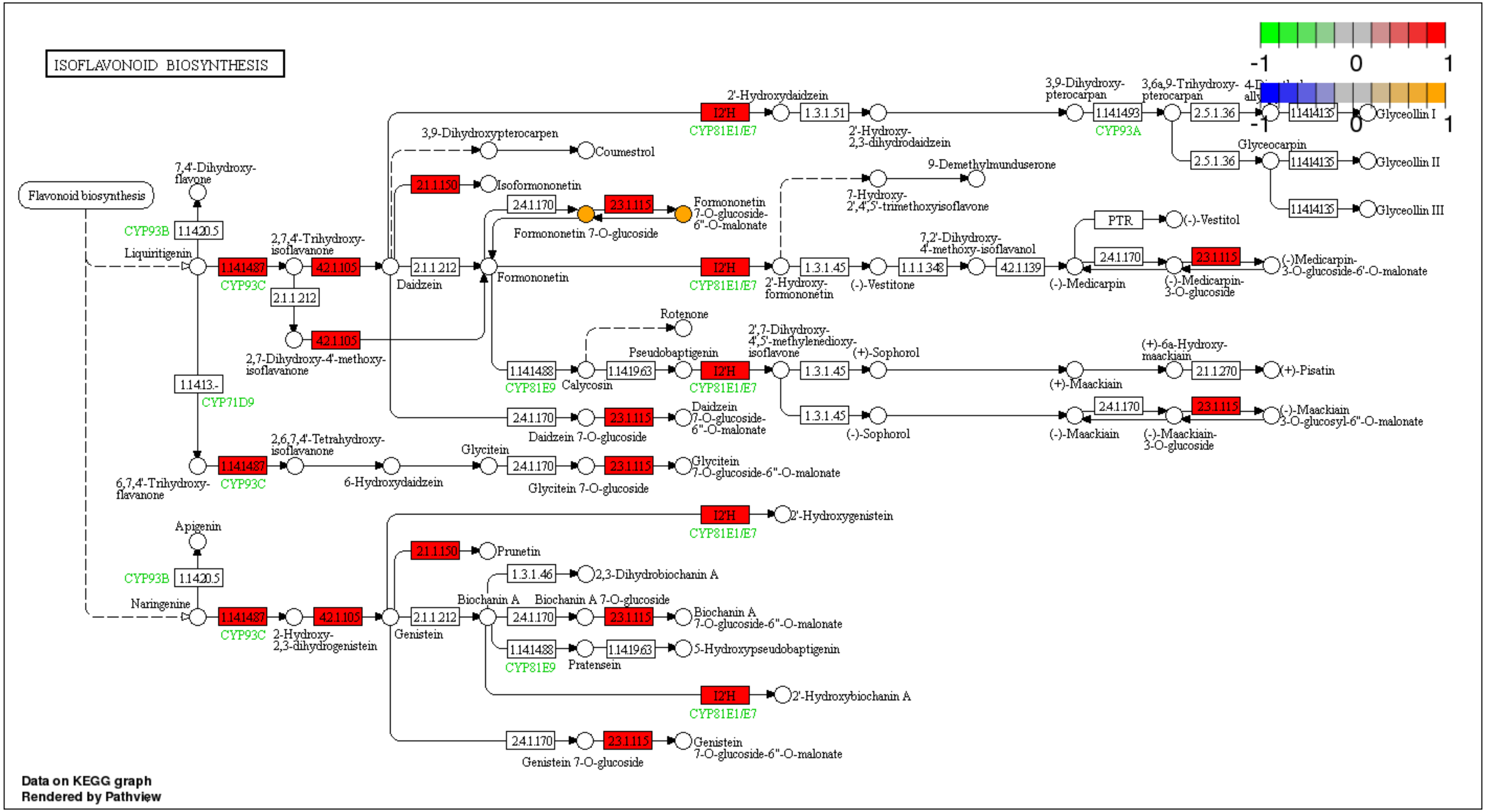
ISOFLAVONOID BIOSYNTHESIS
-1
0
1
-1
0
1
Flavonoid biosynthesis
7,4'-Dihydroxy-flavone
CYP93B
1.14.20.5
Liquiritigenin
1.14.14.87
CYP93C
2,7,4'-Trihydroxy-isoflavanone
4.2.1.105
2.1.1.212
2,7-Dihydroxy-4'-methoxy-isoflavanone
4.2.1.105
Daidzein
2.1.1.212
Formononetin
2.1.1.150
Isoformononetin
3,9-Dihydroxypterocarpen
Coumestrol
2.4.1.170
2.3.1.115
Formononetin 7-O-glucoside
Formononetin 7-O-glucoside-6''-O-malonate
I2'H
CYP81E1/E7
2'-Hydroxydaidzein
1.3.1.51
2'-Hydroxy-2,3-dihydrodaidzein
3,9-Dihydroxy-pterocarpan
1.14.14.93
CYP93A
3,6a,9-Trihydroxy-pterocarpan
2.5.1.36
1.14.14.135
Glyceollin I
Glyceocarpin
Glyceollin II
Glyceollin III
9-Demethylmunduserone
7-Hydroxy-2',4',5'-trimethoxyisoflavone
2'-Hydroxy-formononetin
1.3.1.45
(-)-Vestitone
1.1.1.348
7,2'-Dihydroxy-4'-methoxy-isoflavanol
4.2.1.139
(-)-Medicarpin
PTR
(-)-Vestitol
(-)-Medicarpin-3-O-glucoside
(-)-Medicarpin-3-O-glucoside-6'-O-malonate
1.14.14.1.170
CYP71D9
1.14.13.-
1.14.14.88
CYP81E9
Calycosin
1.14.19.63
Pseudobaptigenin
Rotenone
2',7-Dihydroxy-4',5'-methylenedioxy-isoflavone
(+)-Sophorol
(+)-Maackiain
(+)-6a-Hydroxy-maackiain
2.1.1.270
(+)-Pisatin
(-)-Sophorol
(-)-Maackiain
(-)-Maackiain-3-O-glucoside
(-)-Maackiain-3-O-glucosyl-6''-O-malonate
Daidzein 7-O-glucoside
Daidzein 7-O-glucoside-6''-O-malonate
2,6,7,4'-Tetrahydroxy-isoflavanone
6,7,4'-Trihydroxy-flavanone
6-Hydroxydaidzein
Glycitein
Glycitein 7-O-glucoside
Glycitein 7-O-glucoside-6''-O-malonate
2'-Hydroxygenistein
Apigenin
Naringenine
2-Hydroxy-2,3-dihydrogenistein
Genistein
Prunetin
1.3.1.46
2,3-Dihydrobiochanin A
Biochanin A
Biochanin A 7-O-glucoside
Biochanin A 7-O-glucoside-6''-O-malonate
Pratensein
5-Hydroxypseudobaptigenin
2'-Hydroxybiochanin A
Genistein 7-O-glucoside
Genistein 7-O-glucoside-6''-O-malonate
Data on KEGG graph
Rendered by Pathview

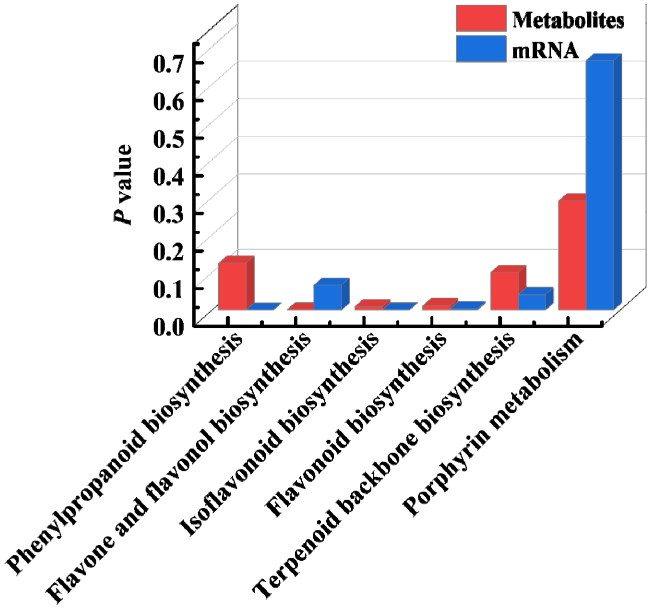

Figure S7. Schematic diagram of the biosynthetic pathway in soybean leaves exposed to 100 mg/L of CDs.

Table S1. The top-50-ranked metabolites based on the ascending *P*-value ranking.

Name	ID	Super Class	Regulation
MFCD24849317	MP6636		Up-regulated
Acexamic acid	MP2248	Lipids and lipid-like molecules	Up-regulated
(2s)-5-hydroxy-2-(4-hydroxyphenyl)-8-{[(2s,3r,4s,5s,6r)-3,4,5-trihydroxy-6-(hydroxymethyl)oxan-2-yl]oxy}-2,3-dihydro-1-benzopyran-4-one	MN1935	Phenylpropanoids and polyketides	Up-regulated
lysilactone A	MN2060		Up-regulated
Iicoisoflavone A 4'-O-glucoside	MN3429		Up-regulated
N-α-L-Acetyl-arginine	MP4166	Organic acids and derivatives	Up-regulated
n-{2-oxa-6-azatricyclo[4.2.1.0³,⁷]nonan-8-yl}carboximidic acid	MP1197	Alkaloids and derivatives	Up-regulated
(5Z,8Z,11S,12R,14Z)-11,12-Dihydroxy-5,8,14-icosatrienoic acid	MP3597		Up-regulated
(1-Phenyl-1H-benzoimidazol-2-ylsulfanyl)acetic acid	MP1544		Up-regulated
Penicillic Acid	MN4036	Organic acids and derivatives	Up-regulated
3,4,2',4',6'-Pentahydroxychalcone 4'-glucoside	MN1379		Up-regulated
1-[(4-Nitrophenyl)amino]-2-pyrrolidinone	MP901		Up-regulated
(2R,3S)-2-[(2S,4R,6E)-4,6-Dimethyl-6-octen-2-yl]-6-oxo-3,6-dihydro-2H-pyran-3-yl (2E,4E,6S)-8-hydroxy-6-(hydroxymethyl)-4-methyl-2,4-octadienoate	MP1835		Up-regulated
3-[(2S,3R,4S,5S,6R)-6-[[(2R,3R,4R)-3,4-dihydroxy-4-(hydroxymethyl)oxolan-2-yl]oxymethyl]-3,4,5-trihydroxyoxan-2-yl]oxy-2-methylpyran-4-one	MN4866	Organic oxygen compounds	Up-regulated
Cyclo(alanylisoleucylprolylleucylleucylserylphenylalanylthreonyl)	MP638		Up-regulated
4-O-[(2E)-3-Phenyl-2-propenoyl]-beta-D-fructofuranosyl alpha-D-glucopyranoside	MN5308		Up-regulated
Coniferol	MN2157	Benzenoids	Up-regulated
4-{[(3S,4R,5S)-3-Hydroxy-5-(4-hydroxy-3-methoxyphenyl)-4-(hydroxymethyl)tetrahydro-3-furanyl]methyl}-2-methoxyphenyl beta-D-glucopyranoside	MP4680	Lignans, neolignans and related compounds	Up-regulated
5,8-Dihydroxy-2-(4-hydroxyphenyl)-7-methoxy-4-oxo-4H-chromen-3-yl 6-deoxy-alpha-L-mannopyranoside	MN2188		Up-regulated

4-Methoxycinnamaldehyde	MP3618	Phenylpropanoids and polyketides	Up-regulated
met-glu-arg	MP3417		Up-regulated
2-(3,4-dihydroxyphenyl)-5,7-dihydroxy-3,4-dihydro-2H-1-benzopyran-4-one	MP2390	Phenylpropanoids and polyketides	Up-regulated
3-oxo-3-{[(2r,3s,4s,5r,6s)-3,4,5-trihydroxy-6-{[3-(4-methoxyphenyl)-4-oxochromen-7-yl]oxy}oxan-2-yl]methoxy}propanoic acid	MN4497	Phenylpropanoids and polyketides	Up-regulated
1-[2,4-dihydroxy-3-[(2S,3R,4R,5S,6R)-3,4,5-trihydroxy-6-(hydroxymethyl)oxan-2-yl]phenyl]-2-hydroxy-3-(4-hydroxyphenyl)propan-1-one	MN664	Phenylpropanoids and polyketides	Up-regulated
Methyl N-(2,3-dihydroxybenzoyl)serinate	MP795		Up-regulated
5,7-Dihydroxy-2-(4-hydroxyphenyl)-4-oxo-4H-chromen-3-yl 6-deoxy-alpha-L-mannopyranosyl-(1-2)-[6-deoxy-alpha-L-mannopyranosyl-(1-6)]hexopyranoside	MN65		Down-regulated
methyl 2-[4-ethenyl-2,6-dihydroxy-3-(3-hydroxyprop-1-en-2-yl)-4-methylcyclohexyl]prop-2-enoate	MP5506	Lipids and lipid-like molecules	Down-regulated
(2R,3R,4S,5S,6R)-2-{[(2E)-4-ethenyl-2,5-dimethylhexa-2,5-dien-1-yl]oxy}-6-(hydroxymethyl)oxane-3,4,5-triol	MP2266		Down-regulated
Gelomulide K	MN7746		Down-regulated
(2R,3S,4S,5R,6R)-2-({[(2R,3R,4R)-3,4-dihydroxy-4-(hydroxymethyl)oxolan-2-yl]oxy}methyl)-6-{[(2E)-3,7-dimethylocta-2,6-dien-1-yl]oxy}oxane-3,4,5-triol	MP2596		Down-regulated
Cyanidin	MP430	Phenylpropanoids and polyketides	Down-regulated
Met-Ile	MP4045	Organic acids and derivatives	Down-regulated
gly-phe-phe	MP2098		Down-regulated
3-{[4,5-dihydroxy-6-(hydroxymethyl)-3-{[(2S,3R,4R,5R,6S)-3,4,5-trihydroxy-6-methyloxan-2-yl]oxy}oxan-2-yl]oxy}-5-hydroxy-2-(4-hydroxyphenyl)-7-{[3,4,5-trihydroxy-6-(hydroxymethyl)oxan-2-yl]oxy}-4H-chromen-4-one	MP30		Down-regulated
terphenyllin	MN1795	Benzenoids	Down-regulated
Melamine	MP106	Organoheterocyclic compounds	Down-regulated

Monomethyl phthalate	MN1625	Benzenoids	Down-regulated
[S-(R*,S*)]-Hexadecanoic acid, 3-[[(2,3-dihydroxypropoxy)hydroxyphosphinyl]oxy]-2-hydroxypropyl ester	MP661	Lipids and lipid-like molecules	Down-regulated
Xanthohumol	MN5225	Phenylpropanoids and polyketides	Down-regulated
trans-Vaccenic acid	MP1901	Lipids and lipid-like molecules	Down-regulated
Linoleoyl ethanolamide	MP1902	Organic nitrogen compounds	Down-regulated
LLK	MP6619		Down-regulated
Rutin	MN439	Phenylpropanoids and polyketides	Down-regulated
Jasmonoyl-L-isoluecine	MP1225	Organic acids and derivatives	Down-regulated
Esculin	MN910	Phenylpropanoids and polyketides	Down-regulated
Cladosporacid D	MN5015	Organic acids and derivatives	Down-regulated
5,7-dihydroxy-2-(4-hydroxyphenyl)-3-{[(2S,3R,4S,5S,6R)-3,4,5-trihydroxy-6-({[(2S,3R,4S,5S)-3,4,5-trihydroxyoxan-2-yl]oxy}methyl)oxan-2-yl]oxy}-4H-chromen-4-one	MP4197		Down-regulated
Pheophorbide A	MP4	Organoheterocyclic compounds	Down-regulated
2-(3,4-Dihydroxyphenyl)-5-hydroxy-4-oxo-4H-chromen-7-yl 6-O-(6-deoxy-alpha-L-mannopyranosyl)-beta-D-glucopyranoside	MN220		Down-regulated
2349_Tetrahydro_Beta_carboline_carboxylic_acid	MP4909	Organoheterocyclic compounds	Down-regulated

Table S2. Up-regulated metabolites with significant differences are listed based on KEGG database ($p < 0.05$).

Pathway	Level 2	Up-regulated metabolites
Flavone and flavonol biosynthesis	Biosynthesis of other secondary metabolites	Luteolin
Isoflavonoid biosynthesis	Biosynthesis of other secondary metabolites	Formononetin-7-O-glucoside<br>Formononetin-7-O-glucoside-6''-O-malonate
Flavonoid biosynthesis	Biosynthesis of other secondary metabolites	Luteolin
Terpenoid backbone biosynthesis	Metabolism of terpenoids and polyketides	-
Phenylpropanoid biosynthesis	Biosynthesis of other secondary metabolites	Coniferol
Biosynthesis of secondary metabolites	Global and overview maps	Benzoic acid<br>Coniferol<br>Luteolin
Porphyrin metabolism	Metabolism of cofactors and vitamins	-
Metabolic pathways	Global and overview maps	Benzoic acid<br>Coniferol<br>Luteolin

Table S3. The top-50-ranked up-regulated and down-regulated genes based on the ascending P-value ranking.

Gene ID	KEGG	EC	Description	Regulation
GLYMA_12G172300	-	-	Uncharacterized protein LOC100817341	Up-regulated
GLYMA_07G090800	-	-	-	Up-regulated
GLYMA_13G077800	-	-	-	Up-regulated
GLYMA_11G142900	-	-	-	Up-regulated
GLYMA_02G222200	-	-	Uncharacterized calcium-binding protein At1g02270 isoform X1	Up-regulated
GLYMA_07G021800	-	-	Chaperone protein dnaJ 8, chloroplastic	Up-regulated
GLYMA_04G066900	K14515	-	EIN3-binding F-box protein 1	Up-regulated
GLYMA_14G116800	K14515	-	EIN3-binding F-box protein 1	Up-regulated
GLYMA_13G140200	-	-	Uncharacterized protein LOC100790091	Up-regulated
GLYMA_13G165600	-	-	Uncharacterized protein LOC100811675	Up-regulated
GLYMA_06G068400	-	-	-	Up-regulated
GLYMA_12G092900	-	-	-	Up-regulated
GLYMA_09G019900	K19042	EC:2.3.2.27	BOI-related E3 ubiquitin-protein ligase 1	Up-regulated
GLYMA_16G044200	K16223	-	Flowering locus T-like protein GmFT3a	Up-regulated
GLYMA_02G303500	-	-	Probable amino acid permease 7 isoform X1	Up-regulated
GLYMA_14G042300	-	-	LOW QUALITY PROTEIN: uncharacterized protein LOC106795977	Up-regulated
GLYMA_13G325800	-	-	Uncharacterized protein LOC100814909	Up-regulated
GLYMA_15G211500	-	-	Alpha-amylase/subtilisin inhibitor	Up-regulated
GLYMA_19G126400	-	-	WAT1-related protein At5g07050	Up-regulated
GLYMA_11G093100	-	-	Isoflavone 3'-hydroxylase	Up-regulated
GLYMA_10G184700	-	-	Putative protease inhibitor	Up-regulated
GLYMA_20G220900	-	-	Cupin family protein precursor	Up-regulated
GLYMA_14G042900	-	-	-	Up-regulated
GLYMA_13G036600	K03875	-	F-box protein SKP2B	Up-regulated

GLYMA_17G023000	-	-	-	Up-regulated
GLYMA_19G076800	-	-	Lysine histidine transporter 1	Up-regulated
GLYMA_04G220600	K00430	EC:1.11.1.7	Peroxidase P7	Up-regulated
GLYMA_15G061400	-	-	Transcription factor bHLH25-like	Up-regulated
GLYMA_03G122200	-	-	-	Up-regulated
GLYMA_04G231400	-	-	-	Up-regulated
GLYMA_16G170100	-	-		Up-regulated
GLYMA_06G141600	-	-	Uncharacterized protein LOC100775497	Up-regulated
GLYMA_01G051300	-	-	NAC domain protein NAC1	Up-regulated
GLYMA_17G239000	-	-	Cysteine proteinase RD21A	Up-regulated
GLYMA_09G054600	-	-	Uncharacterized protein LOC100803921 isoform X2	Up-regulated
GLYMA_08G235400	-	-	-	Up-regulated
GLYMA_13G189700	-	-	Uncharacterized protein LOC100804390	Up-regulated
GLYMA_18G281400	-	-	-	Up-regulated
GLYMA_12G059100	K08081	EC:1.1.1.206	Tropinone reductase	Up-regulated
GLYMA_05G124900	K22395	EC:1.1.1.195	Berberine bridge enzyme-like 28	Up-regulated
GLYMA_06G112200	-	-	WAT1-related protein At4g08300	Up-regulated
GLYMA_02G109800	-	-	-	Up-regulated
GLYMA_08G235300	-	-	-	Up-regulated
GLYMA_13G278000	-	-	-	Up-regulated
GLYMA_09G048700	K13260	-	Isoflavone 2'-hydroxylase	Up-regulated
GLYMA_01G137700	K01051	EC:3.1.1.11	Pectinesterase 2	Up-regulated
GLYMA_14G156400	K18857	EC:1.1.1.1	Alcohol dehydrogenase 1	Up-regulated
GLYMA_10G005400	-	-	Protein NRT1/ PTR FAMILY 5.2	Up-regulated
GLYMA_07G251300	-	-	-	Up-regulated
GLYMA_01G169200	K09755	EC:1.14	Cytochrome P450 84A1-like	Up-regulated

GLYMA_10G224500	-	-	Protein HLB1	Down-regulated
GLYMA_19G174200	-	-	-	Down-regulated
GLYMA_09G072000	-	-	Ethylene-responsive transcription factor ERF017	Down-regulated
GLYMA_04G226700	-	-	-	Down-regulated
GLYMA_17G030700	-	-	COBRA-like protein 7	Down-regulated
GLYMA_06G138100	-	-	NAC domain-containing protein 91	Down-regulated
GLYMA_02G245700	K13448	-	Probable calcium-binding protein CML18	Down-regulated
GLYMA_17G034900	-	-	-	Down-regulated
GLYMA_07G266500	-	-	Uncharacterized protein LOC100792931	Down-regulated
GLYMA_02G212100	-	-	-	Down-regulated
GLYMA_17G047300	-	-	Dehydration-responsive element binding protein 3	Down-regulated
GLYMA_13G112400	-	-	Ethylene-responsive transcription factor ERF017	Down-regulated
GLYMA_14G220400	-	-	Reticulocyte binding protein 2 homolog b isoform X2	Down-regulated
GLYMA_13G352200	K12197	-	ESCRT-related protein CHMP1B	Down-regulated
GLYMA_09G031700	-	-	-	Down-regulated
GLYMA_01G183300	-	-	Probable disease resistance protein At5g66900	Down-regulated
GLYMA_13G285400	-	-	Chitin-inducible gibberellin-responsive protein 1	Down-regulated
GLYMA_04G136600	-	-	-	Down-regulated
GLYMA_04G092100	K15104	-	Mitochondrial uncoupling protein 4	Down-regulated
GLYMA_11G137300	-	-	E3 ubiquitin-protein ligase RDUF2	Down-regulated
GLYMA_11G243000	K00737	EC:2.4.1.144	Uncharacterized protein LOC100780085	Down-regulated
GLYMA_13G069700	-	-	-	Down-regulated
GLYMA_06G036300	-	-	Serpin-ZX	Down-regulated
GLYMA_06G089800	K00913	-	Inositol phosphate kinase	Down-regulated
GLYMA_05G079700	K02183	-	Calmodulin	Down-regulated
GLYMA_14G171500	K09286	-	Ethylene-responsive transcription factor RAP2-4	Down-regulated

GLYMA_02G132500	-	-	Ethylene-responsive transcription factor ERF109	Down-regulated
GLYMA_15G180000	-	-	Ethylene-responsive transcription factor ERF017	Down-regulated
GLYMA_11G194600	-	-	-	Down-regulated
GLYMA_13G294200	-	-	Uncharacterized protein LOC100820571	Down-regulated
GLYMA_14G041700	-	-	NDR1/HIN1-like protein 13	Down-regulated
GLYMA_14G195200	K17506	EC:3.1.3.16	Probable protein phosphatase 2C 25	Down-regulated
GLYMA_08G015700	-	-	BTB/POZ domain-containing protein At5g41330	Down-regulated
GLYMA_02G040700	K15104	-	Mitochondrial uncoupling protein 5	Down-regulated
GLYMA_14G179800	-	-	Uncharacterized protein LOC100802817	Down-regulated
GLYMA_07G212400	-	-	-	Down-regulated
GLYMA_14G106200	-	-	-	Down-regulated
GLYMA_10G266900	-	-	Sarcoplasmic reticulum histidine-rich calcium-binding protein	Down-regulated
GLYMA_13G088100	K09286	-	Ethylene-responsive transcription factor RAP2-4	Down-regulated
GLYMA_16G178400	-	-	Heavy metal-associated isoprenylated plant protein 12	Down-regulated
GLYMA_12G119200	-	-	Thioredoxin-like superfamily protein	Down-regulated
GLYMA_08G045200	K07766	EC:3.6.1.52	Nudix hydrolase family protein	Down-regulated
GLYMA_07G087200	-	-	-	Down-regulated
GLYMA_17G259300	-	-	Kinesin-related protein 12	Down-regulated
GLYMA_01G216000	-	-	Dehydration-responsive element-binding protein 1E	Down-regulated
GLYMA_20G123800	-	-	Protein FAM133	Down-regulated
GLYMA_11G144600	K07195	-	Exocyst complex component EXO70H1	Down-regulated
GLYMA_16G016300	-	-	Uncharacterized calcium-binding protein At1g02270 isoform X2	Down-regulated
GLYMA_04G208300	-	-	-	Down-regulated
GLYMA_06G124300	-	-	Dof zinc finger protein DOF3.5	Down-regulated

Table S4. Genes with significant differences are listed based on KEGG database.

Gene ID	KEGG	EC	Description	Regulation
GLYMA_09G019900	K19042	EC:2.3.2.27	BOI-related E3 ubiquitin-protein ligase 1	Up-regulated
GLYMA_04G220600	K00430	EC:1.11.1.7	Peroxidase P7	Up-regulated
GLYMA_12G059100	K08081	EC:1.1.1.206	Tropinone reductase	Up-regulated
GLYMA_05G124900	K22395	EC:1.1.1.195	Berberine bridge enzyme-like 28	Up-regulated
GLYMA_01G137700	K01051	EC:3.1.1.11	Pectinesterase 2	Up-regulated
GLYMA_14G156400	K18857	EC:1.1.1.1	Alcohol dehydrogenase 1	Up-regulated
GLYMA_01G169200	K09755	EC:1.14	Cytochrome P450 84A1-like	Up-regulated
GLYMA_11G243000	K00737	EC:2.4.1.144	Uncharacterized protein LOC100780085	Down-regulated
GLYMA_14G195200	K17506	EC:3.1.3.16	Probable protein phosphatase 2C 25	Down-regulated
GLYMA_08G045200	K07766	EC:3.6.1.52	Nudix hydrolase family protein	Down-regulated
